# Cellular Immune Response after Vaccination in Patients with Cancer—Review on Past and Present Experiences

**DOI:** 10.3390/vaccines10020182

**Published:** 2022-01-25

**Authors:** Maria Madeleine Rüthrich, Nicola Giesen, Sibylle C. Mellinghoff, Christina T. Rieger, Marie von Lilienfeld-Toal

**Affiliations:** 1 Department of Internal Medicine II, Hematology and Medical Oncology, Universitätsklinikum Jena, Am Klinikum 1, 07747 Jena, Germany; maria.ruethrich@med.uni-jena.de; 2 Leibniz Institute for Natural Product Research and Infection Biology, Hans-Knöll-Institut, Adolf-Reichwein-Straße 23, 07745 Jena, Germany; 3 Department of Haematology and Oncology, Internal Medicine V, University Hospital Heidelberg, 69115 Heidelberg, Germany; nicola.giesen@med.uni-heidelberg.de; 4 Centre for Integrated Oncology Aachen Bonn Cologne Düsseldorf (CIO ABCD), Faculty of Medicine and University Hospital of Cologne, Department I of Internal Medicine, University of Cologne, 50923 Cologne, Germany; sibylle.mellinghoff@uk-koeln.de; 5 Hemato-Oncology Germering & Interdisciplinary Tumorcenter, Ludwig-Maximilians-University Munich, 81377 Munich, Germany; christina.rieger@med.uni-muenchen.de

**Keywords:** vaccination, cellular response, SARS-CoV-2, cancer, malignancy

## Abstract

Patients with cancer are at particular risk for infection but also have diminished vaccine responses, usually quantified by the level of specific antibodies. Nonetheless, vaccines are specifically recommended in this vulnerable patient group. Here, we discuss the cellular part of the vaccine response in patients with cancer. We summarize the experience with vaccines prior to and during the SARS-CoV-2 pandemic in different subgroups, and we discuss why, especially in patients with cancer, T cells may be the more reliable correlate of protection. Finally, we provide a brief outlook on options to improve the cellular response to vaccines.

## 1. Introduction

Vaccination is indispensable for infection control as has been demonstrated in the SARS-CoV-2 pandemic. Patients with malignancies are at particular risk for infection and vaccination is explicitly recommended [1,2], but humoral response to vaccination is often impaired due to disease and treatment. In contrast, cellular response, in particular T cellular response, has recently been recognized to be generally more robust than humoral response and possibly even more predictive for protection [3,4]. A literature search on the experience with vaccines prior to and during the SARS-CoV-2 pandemic in patients with cancer was performed. Search sources were the electronic databases MEDLINE/PubMed and Google Scholar; key search terms included “T cell response”, “cellular immune response”, “cancer”, “malignancy”, “SARS-CoV-2”, and “vaccination”. Relevant content was extracted and revised on the basis of an email-based discussion process. 

In this review, we will describe what is known about the T cellular response to different types of vaccines in cancer patients, delineate the influence of certain types of cancer and/or the respective treatment, and finally, summarize what is currently known regarding the cellular response of patients with malignancies towards SAR-CoV-2 vaccines. 

## 2. Characteristics of Cellular Vaccine Response

### 2.1. T Cell Populations Involved in Vaccine Response

Several types of T lymphocytes are deemed to play relevant roles in the cellular defense against vaccine-preventable infections [5,6]. These include antigen-specific CD8+ T cells, which kill human cells infected by the pathogen via release of cytotoxic enzymes such as granzyme B. Similarly, antigen-specific CD4+ T cells with the ability to kill infected cells have been described [7]. In addition, CD4+ T helper cells further support antibody production and cytokine secretion.

In general, the cellular response to vaccination is more robust and more reliably induced in vulnerable populations such as the elderly [3] or patients with comorbidities, even including patients without B cells [8], than the humoral response. Although the duration of T cell-induced protection is very variable and can be as short as six months [9], durable responses as long as three years after vaccination with an inactivated herpes zoster (HZ) vaccine have been described [10].

In addition, T cells are more likely to be cross-reactive to strains different from the vaccination strain or strains that caused a prior infection. This phenomenon was observed during the influenza H1N1 pandemic [11] in experimental set-ups of influenza infection [12], as well as recently in COVID-19 [13]. In the SARS-CoV-2 pandemic, recent data showed a reduced humoral immune response to variants of concern, in particular to delta, whereas T cell response did not differ, suggesting a cell-mediated protection from severe disease [8,14]. However, for the very recently emerged variant of concern, Omicron, this has yet to be confirmed. Generally, cross-reactive T cell responses may be induced and maintained by repeated vaccination [15].

### 2.2. Risk Factors for Reduced T Cell Vaccine Response

Most risk factors that have been reported to be associated with a reduced cellular response to vaccination are linked to immunosenescence. It is well known that older people develop reduced vaccine efficacy, and this has recently been confirmed in the COVID-19 vaccine trials [16,17,18]. Not surprisingly, similar effects have also been observed in cancer patients, both in serological [19] and clinical studies [20]. However, additional factors are likely to contribute to a reduced vaccine response. One of those is a reduction in the naïve T cell pool, contributing to a failure to expand clones on demand. This may be due to clonal expansions of memory T cells at the cost of naïve T cells. The majority of these memory T cells are CD8+ CD25−, which are often virus specific and have been associated with poor response to vaccination (for example in CMV persistence) [21,22,23].

### 2.3. Assessment of T Cell Vaccine Response 

Several methods are commonly used to assess the cellular response to vaccination induced by T cells [5]. These include the quantification and characterization of pathogen-specific T cells as well as the estimation of the T cell function by cytokine measurement. For enumeration of antigen-specific T cells, flow-cytometrical approaches using tetramer-staining are commonly employed. This facilitates the assessment of the antigen-specific T cell activation levels by analyzing the co-expression of activation markers such as CD25 or CD40L. Additionally, cytotoxic activity can be measured on the cellular level by CD107a upregulation, and functionality can be assessed using intracellular cytokine staining (e.g., interferon gamma (IFNγ), interleukin-2 (IL-2), or tumor necrosis factor-α (TNFα)). Another method to measure the cytokine response on a single cell basis is the ELISPOT technique (typically IFNγ ELISPOT). This can be advanced by the fluorospot technique, which can analyze up to three analytes on one cell. On a broader level, cytokine concentrations can be measured in soluble samples. Here, platforms with >10 analytes are often implemented in modern laboratories. Novel techniques further use Omics approaches such as RNA-seq to detect up- or downregulation of proteins in activated T cells.

As most of these techniques are labor-intensive and time-consuming, few assays have found their way into clinical practice. By far the most frequently used test for vaccine induced T cell response is the IFNγ ELISPOT, which is reported by most diagnostic trials involving large patient populations [8,24,25,26].

### 2.4. T Cells as Correlates of Protection

An important issue is the correlation of the cellular response to vaccination with protection against vaccine-preventable infection and infectious disease. In a very early experimental trial, the presence of cytotoxic T cells elicited by prior infection was found to be more protective of influenza disease than the presence of antibodies. Additionally, T cells were found to be cross-reactive between strains and thus less susceptible to antigenic drift [12]. Another experimental trial inducing influenza infection in sero-negative healthy individuals confirmed the protective effect of pre-existing T cells (T helper cells in particular) on the duration and severity of symptoms [27]. These findings are supported by clinical observations: in a prospective diagnostic study during one influenza season comprising mostly elderly people, post-vaccination antibody titers were less useful in predicting protection from influenza infection than the induction of a cellular cytokine response [3]. Additionally, the protective role of cross-reactive T cells in naturally sero-negative healthy individuals on the severity of illness could be confirmed during the influenza H1N1 pandemic in 2009/2010 [11].

Data regarding T cells as correlates of protection in cancer patients are still scarce. However, there are indirect data supporting a significant role of cellular vaccine response in cancer patients. In several recent diagnostic studies, patients with hematological malignancies showed markedly reduced serological responses to the messenger ribonucleic acid (mRNA) vaccines against COVID-19 compared to patients with solid tumors [8,19,24]. Yet, despite these notable differences in serological vaccine response, the clinical vaccine efficacy of mRNA vaccines against COVID-19, as shown in a large clinical study comparing more than 20,000 immunocompromised with more than 60,000 immunocompetent people, was rather similar between patients with hematological malignancies and those with solid tumors (74% and 79%, resp.) [20]. Thus, it is conceivable that the cellular vaccine response may actually be more predictive as a correlate of protection in patients with cancer, similar to the observations made in the geriatric population [3].

#### Overview of Vaccines Used in Cancer Patients

Patients with malignancies are known to be at an increased risk for severe infections since the underlying malignancy and its treatment, ranging from steroids to hematopoietic cell transplantation (HCT), can cause profound suppression of both cellular and humoral immune responses [28,29,30]. Consequently, limitations in vaccine efficacy and immunogenicity are to be expected. However, while many studies report on the humoral responses to vaccination, much less is known about individual vaccine induced cell-based responses in immunocompromised patients [29,31,32,33,34]. Notably, recent publications suggest an important role for T cells [35,36,37,38], although the extent depends on the underlying disease and treatment.

Vaccines licensed for human use can be categorized according to the vaccine platform technology used for development. Classical platforms include live-attenuated pathogens (e.g., measles, rubella, mumps), inactivated pathogens (e.g., polio, first influenza vaccines), and (viral) protein platforms (e.g., influenza, human papillomavirus, hepatitis B), while new approaches, so-called next-generation vaccine platforms, are based on genome sequence information [39,40,41] and include mRNA vaccines as well as vector-based vaccines.

### 2.5. Cellular Responses Induced by Classical Vaccines

Live-attenuated vaccines consist of pathogens weakened by repetitive passages through non-human cell cultures. Mimicking natural infection, attenuated pathogens elicit a strong cellular and humoral immune response without causing a severe disease in healthy individuals [2,39,42,43]. In contrast, live-attenuated vaccines are contraindicated for use in immunocompromised individuals, as they have the capacity to uncontrolled replication and reversion to the wild phenotype, potentially causing lethal disease [2,44]. Inactivated pathogen vaccines are inactivated by irradiation, chemicals, or heat and are therefore less immunogenic when compared to live-attenuated vaccines. Here, the addition of an external adjuvant and booster vaccinations are ways to elicit a sufficiently strong immune response, even in immunocompetent individuals [39,45,46]. (Viral) protein-based vaccine platforms comprise subunit, split virus vaccines, and virus-like particles (VLP). They are developed by protein isolation and purification or recombinant synthesis [45]. While subunit protein vaccines contain surface glycoproteins, split virus vaccines contain both surface and internal proteins. Following vaccination, the pathogen is processed by antigen presenting cells (APC) and presented to adaptive immune cells inducing a humoral and cellular response [46]. VLP are empty virus particles presenting numeric copies of key viral structure proteins on their surface, inducing a strong antigen-specific immune response by interacting directly with APC [45]. Seasonal trivalent or quadrivalent influenza vaccines are protein-based and may contain either subunit products or split-virus products. While the humoral immune response is reported to be comparable following subunit or split virus vaccination, cellular immune response seems to be stronger in case of split virus vaccines [47,48,49]. Regarding SARS-CoV-2, several vaccines based on classical platforms already entered phase II/III clinical trials or in approval phase. Novavax is the first of these classical platform-based COVID-19 vaccines that received both approval by the Food and Drug Administration (FDA) and the European Medicines Agency (EMA). It is a recombinant subunit protein vaccine containing the adjuvant Matrix M1, which is known to stimulate the cell-mediated immune system [50,51]. A CD4+ T cell activation was measurable in all tested individuals, while the addition of M1 enhanced cell-mediated immune response with a strong bias towards a Th 1 phenotype [45,51]. Another of these classical vaccines is CoronaVac, an inactivated virus, alum-adjuvanted, vaccine. In addition to a humoral immune response, cellular immunogenicity was seen in the majority of recipients. Notably, in individuals without a measurable number of neutralizing antibodies, virus specific memory CD4+ and CD8+ T cells were detected [52]. A recently published phase I open-label trial on CoVac-1, a peptide-based vaccine, showed a SARS-CoV-2 specific CD4+, namely Th 1 phenotype, and CD8+ T cell response. To stimulate a stronger cellular immune response, an adjuvant (Montanide ISA 51—a water–oil emulsion) was added [53]. A plant-produced virus-like particle vaccine (CoVLP) was introduced by Ward et al., demonstrating both a humoral and cellular immunogenicity. Of note, again, immunogenicity was enhanced using an adjuvant [54]. 

### 2.6. Cellular Responses Induced by Nucleic Acid Vaccines 

Nucleic acid vaccine platforms consist of deoxyribonucleic acid (DNA) or ribonucleic acid (RNA), encoding the vaccine antigen [39]. For subsequent processing, DNA vaccines must enter the nucleus, while mRNA vaccines only need to penetrate the cellular membrane [39,45]. Following processing of mRNA, antigens are presented to adaptive immune cells [45]. By now, two mRNA vaccines are licensed by FDA/EMA for human use, BNT162b2 and mRNA-1273, both known to elicit humoral and cellular immunogenicity in healthy individuals [33,55,56,57]. Upon vaccination with BNT162b2, a strong response of virus-specific Th1 and CD8+ T cells was reported, in some of them more than tenfold compared to responses to common viruses [33,56,57]. In individuals who received mRNA-1273, a strong CD4+ cytokine response involving Th1 cells was seen. In line with this, the response involving virus-specific Th 2 cells was only minimal [58]. A T cell response dominated by CD8+ T cells expressing both IFNɣ and TNFα was shown by preliminary data from a phase I trial on 40 healthy individuals who received INO-4800—a SARS-CoV-2 DNA vaccine [59]. Following official approval, several research groups assessed humoral and cell-mediated immune responses following mRNA vaccination in cancer patients. One common feature was a discordance in humoral and cellular immune response in specific patient groups. Whereas in the majority of immunocompetent patients the humoral and the T cell vaccine response correlated well, in B cell depleted patients a drastically reduced antibody response was observed, while about a third of these patients still had specific T cells. In contrast, patients after HCT showed a more pronounced reduction in T cellular response whilst preserving the antibody response [8,29,31,32,60,61,62]. Relevant factors impacting T cell vaccine-induced immune response beyond humoral response and vice versa are yet to be determined in studies of larger sample sizes.

Viral vector vaccines consist of an attenuated recombinant virus (vector virus), expressing genes encoding for specific epitopes [63]. Common vectors are adenovirus, measles, and vesicular stomatitis virus (VSV) [45]. Viral vector vaccines can induce robust humoral and cellular responses with a single dose. Notably, pre-existing immunity against a human viral vector can weaken the immune responses [45,64]. The first viral vector vaccine was approved in 2019. It is based on a recombinant VSV vector that contains the genetic information codifying for an Ebola glycoprotein [65]. With the SARS-CoV-2 pandemic, further viral vector vaccines were approved. All of them elicit specific CD8+ and Th1-biased CD4+ T cell responses in healthy individuals [64,66,67,68].

## 3. Cellular Immune Response to Vaccination with Classical Vaccines in Patients with Cancer—Special Patient Populations and Therapeutic Subgroups

### 3.1. Patients with Hematological Malignancies

Regarding the annual inactivated influenza vaccine, a vaccine-induced T cell response has been described. In a small cohort of 13 patients with myeloproliferative neoplasms (MPN), the percentages of naïve and active CD4+ T cells were measured prior, three weeks following, and three months following vaccination. Compared to healthy individuals, both naïve and active CD4+ T cells were significantly lower at baseline. In contrast, after three weeks, the number of naïve CD4+ T cells and, after three months, the number of active CD4+ T cells were significantly higher compared to healthy individuals, suggesting a delayed cellular immune response in this patient population [69]. Another study showed a similar T cell response increasing from day 50 in patients with hematological malignancies compared to healthy individuals [70]. 

### 3.2. High-Dose Therapy and Stem Cell Transplantation

Cellular immunogenicity of inactivated vaccines in patients with malignancies is best described in patients who underwent HCT. In the post-engraftment period following HCT, a marked decrease in cellular immunity is observed, most severely in allogeneic HCT patients [71,72]. T cell reconstitution after allogeneic HCT requires several months, especially in older patients and after myeloablative conditioning. It may be further hampered by graft-versus-host disease (GVHD) and immunosuppressive treatment [72]. Vaccination is therefore usually not recommended until at least six months after allogeneic HCT using inactivated vaccines and until at least two years using live vaccines [2,73]. In contrast, after autologous HCT, this long pause may not be necessary, as suggested by the data from the following studies.

For pneumococcal vaccination Locke et al. demonstrated that the conjugate vaccine Prevenar-13 (PCV-13), administered to patients with multiple myeloma before G-CSF mobilization and during the lymphopenic period up to 21 days after autologous HCT, elicited an increase in T helper cells and intracellular IFNγ and cytotoxic T cells [74]. A phase I/II observer-blind, randomized, and placebo-controlled trial investigated an adjuvanted recombinant subunit HZ vaccine in autologous HCT recipients. Cell-mediated immune response was measured by antigen-specific CD4+ T cell frequency. Compared to the control group (saline), cellular immune response was significantly higher in vaccinated patients. Notably, three doses was superior to two vaccine doses [75]. Other studies showed that heat-inactivated varicella zoster virus (VZV) vaccines in adult autologous HCT recipients elicited early recovery of T cell responses to the virus. Concomitantly, expression of INFγ, TNFα, and IL-10 was significantly higher among patients with virus-specific T cell proliferation [76,77]. The cellular immunogenicity correlated with a reduced risk of HZ [76]. Thus, a strong cellular response to vaccination seems to be achievable even relatively early after autologous HCT.

This strategy on early vaccination after autologous HCT was further pursued with recombinant VZV subunit or inactivated VZV vaccines [75,78,79,80]. The ZOE-HSCT randomized, placebo-controlled phase 3 trial demonstrated efficacy of a two-dose regimen of a recombinant VZV vaccine against VZV glycoprotein E administered 50–70 days after autologous HCT against development of HZ [80]. Cellular vaccine response, assessed by frequency of glycoprotein E specific CD4+ T cells expressing at least two activation markers, was achieved in 93% of patients in the vaccine group at one month after the second dose and declined to 71% after two years, while no increase in activated CD4+ T cells was observed in the placebo cohort post vaccination compared to prior to vaccination [80]. The V212-001 phase 3 trial evaluated a four-dose regimen of inactivated VZV vaccine administered prior as well as 30, 60, and 90 days after autologous HCT [78]. Cellular response to vaccination, assessed by IFNγ enzyme-linked immunospot assay, showed a ratio of estimated geometric mean fold rise (GMFR) in vaccine versus placebo group of 5.41 at four weeks after dose 4 and 3.32 at two years [79]. Compared to baseline, a GMFR of 1.85 was observed in vaccine recipients at four weeks after dose 4, which increased to 3.32 at two years, while estimated geometric mean count in the placebo group was actually lower at four weeks after dose 4 compared to baseline [79].

In a study investigating a two-dose regimen of inactivated H1N1 influenza vaccine in adult hematological patients, induction of a significant H1N1 specific T cell response was observed in the subgroup of allogeneic HCT patients [81]. Interestingly, T cell response did not differ significantly between patients and healthy controls [81].

### 3.3. Cellular Therapy and Monoclonal Antibodies

B cell depletion following anti-CD20 or anti-CD19 directed therapies, such as anti-CD20 monoclonal antibodies, anti-CD19 antibody drug conjugates (ADCs), or anti-CD19 chimeric antigen receptor (CAR) T cells, is generally assumed to impede an adequate response to vaccination. Current guidelines recommend forgoing vaccination until B cell recovery [2], as this is known to lead to dramatically reduced serological responses after vaccination [19,82]. In contrast, cellular response is less well studied in this patient population. Regarding protein- or polysaccharide-based inactivated vaccines, a small study evaluated response to vaccination with *haemophilus influenzae* conjugate vaccine and pneumococcal polysaccharide vaccine in patients with immune thrombocytopenia treated with rituximab versus placebo 6 months earlier [83]. Compared to the placebo cohort, significantly fewer INFγ-secreting T cells were observed in the rituximab cohort 4 weeks after vaccination. However, the T cell response was more robust than the antibody response, which was missing in about 70% of patients after rituximab [83].

A small clinical trial on immune response to vaccination with an inactivated VZV vaccine in patients with hematological malignancies treated with anti-CD20 monoclonal antibodies demonstrated a significant VZV-specific cellular immune response after dose 4, as measured by INFγ enzyme-linked immunospot [26]. In a cross-trial comparison to patients with hematological malignancies without anti-CD20 monoclonal antibodies, the strength of cellular immune response between the two patient populations was comparable [26,84].

In summary, a cellular immune response to vaccination can be achieved in B cell depleted patients, which seems to be decreased compared to healthy individuals but similar when compared to patients with hematological malignancies on other treatment regimens. Regarding the more recently approved plasma cell directed therapies, such as anti-CD38 monoclonal antibodies, anti-BCMA ADCs or bispecific antibodies, or anti-BCMA CAR T cells, far less is known on their impact on vaccine response, and most data come from recent studies analyzing response after COVID-19 vaccination, as described below.

### 3.4. Anti-Cancer Treatments: Chemotherapy, Immune Checkpoint Blockade and Small Molecules

The cellular response in patients undergoing chemotherapy was investigated in a phase II/III study including 232 patients with solid cancer who received an adjuvant recombinant HZ vaccine. In treatment-naïve patients, the first dose was scheduled 8 to 30 days before, and in patients currently under anticancer therapy, at the start of a treatment cycle. The second dose was administered with a subsequent chemotherapy cycle. An increase in antigen-specific CD4+ T cells was seen in both groups compared to placebo. Notably, the humoral response was higher in treatment-naïve patients [85]. In solid tumor patients receiving immune checkpoint inhibitors, vaccine-induced expression of H1N1-specific CD4+ and CD8+ T cells were more frequent compared to patients under conventional cytotoxic chemotherapy [86]. A further analysis on influenza vaccines included patients with metastatic renal cell cancer or GIST and healthy participants. Cellular immune response was measured at baseline and day 8. The majority of patients were treated with tyrosine kinase inhibitors (i.e., sunitinib or sorafenib). Functional T cell response was observed in all patients with the exception of a lower concentration of IFNɣ in patients treated with sorafenib [87]. Thus, little influence on the cellular immune response seems to be exerted at least by these types of small molecules.

## 4. Special Situation: Cellular Response to Vaccination against SARS-CoV-2 in Patients with Cancer

### 4.1. Patients with Hematological Malignancies

While most previous vaccination studies in cancer patients have mainly focused on serologic data [88,89,90,91], few also describe cellular responses to determine immunogenicity [92]. Regarding COVID-19 vaccines, several reports describe the dynamics of binding anti-SARS-CoV-2 antibodies. They observed a reduced serologic response in hematological patients [8,31,34,82,93,94,95]. The observed poor immunogenicity induced by COVID-19 vaccines in hematological patients is in line with other vaccines in this population such as hepatitis B, influenza, and pneumococcal vaccines.

A defined correlate of protection is still lacking for COVID-19 vaccines, but there are strong indications towards neutralizing antibodies as such against SARS-CoV-2 infection [96,97,98]. Yet, it is questionable if the serologic response can serve as reliable surrogate in a population with an impaired B cell axis.

As previous investigations in hematological and B-cell depleted patients revealed, the T cell response to SARS-CoV-2 infection and COVID-19 vaccination plays a vital role and may ensure protection even in the absence of either humoral or B cell response [35,60]. Agammaglobulinemia patients were shown to recover from COVID-19 without adequate serological responses, suggesting a T cell response to be sufficient for recovery from disease and even for mounting protection from infection [99,100]. An undetectable B cell and reduced T cell response in many hematological patients (ranging from 34.2 to 79.0%) compared to healthy controls or solid tumor patients has so far been reported [31,61,62,101,102]. Age (>65 years), active disease, immunosuppressive treatment for GvHD, and lymphopenia were associated with impaired cellular response.

Especially in patients treated with B cell depleting treatment, a significant dissociation between humoral and cellular response was observed, as many of those lack humoral response but were shown to have a SARS-CoV-2 specific T cell vaccine-induced immune response. In contrast, patients with immunosuppressive treatment (e.g., for GvHD) tend to have a stronger humoral, but reduced cellular response [62].

Few data suggest that heterologous vaccination [103] and a booster immunization could enhance T cell response especially in immunocompromised patients. It might thus be a reasonable approach to adapt the vaccination strategy for hematological patients and those with otherwise caused B cell depletion. It is reassuring that a high vaccine-induced T cell response is reported in immunocompromised patients, even if they fail to seroconvert. The underlying mechanism is yet to be determined. Regulatory B cells and antigen abundance due to antibody absence may subsequently modulate T cell activation and proliferation [104]. A more comprehensive picture by in-depth T cell analyses in this population may grant further insights.

### 4.2. Solid Malignancies

While hematological diseases are associated with impaired humoral and—partly—cellular vaccine immune responses, this is not necessarily the case for patients with solid malignancies. In a cohort of 180 patients with solid cancer, 90% of patients exhibited an adequate antibody response to the BNT162b2 vaccine, although their amounts of antibody titers were significantly lower than those of healthy controls [105]. Of note, 75% had metastatic disease demanding excessive treatment. Yet, further studies of similar size and with different distribution of solid cancer location showed lower seroconversion rates compared to healthy controls [106,107]. As the serological vaccine immune response depends on underlying disease and type of treatment, the same may be assumed for cellular response. The latter was shown to be impaired in many patients with solid cancer (46%) in a recent observation [31]. Although more recent studies describe higher rates (up to 89.5%) of achieved vaccine-induced cellular responses, they also describe substantially reduced magnitudes of vaccine-induced antibody and T cell responses in patients with cancer compared to healthy individuals [34,61]. Depending on the underlying disease and type of treatment, cancer patients also exhibited discordant immune responses and may have a T cell response even if failing seroconversion.

Of note, the third immunization may improve the cellular immune response to the vaccine [108], although this is considered controversial [34]. In a situation of increasing COVID-19 case numbers and novel variants of concern, this must be investigated in studies of larger scope to conclude clinical implications.

### 4.3. High Dose Therapy and Stem Cell Transplantation

In a prospective cohort study investigating safety and immunogenicity of the COVID-19 BNT162b2 vaccine in allogeneic transplant patients, 75% of patients, all at least 3 months after HCT and without severe GVHD, showed a serologic response, while a cellular vaccine response was only achieved in 19% of evaluable patients [62]. A positive correlation was observed between a higher CD4+/CD8+ ratio and a cellular response, although statistical power was hampered by small sample size [62]. Similarly, in a large cohort study on more than 100 allogeneic HCT patients vaccinated with two doses of BNT162b2 at a median of 30 months following transplant, cellular response to vaccination was significantly reduced [109]. The time interval between HCT and vaccine response assessment positively correlated with both humoral and cellular response [109]. In contrast, a smaller prospective study analyzing T cell responses to sequential COVID-19 vaccination, mostly mRNA-based, in allogeneic HCT patients reported a significantly positive impact of repeat vaccination with an increase in T cell response from only 35% of patients (after the first dose) to 82% (after the second dose) [110].

### 4.4. Cellular Therapy and Monoclonal Antibodies

Recent observations in COVID-19 vaccine response in patients with chronic lymphatic leukemia confirm the absence of development of (mRNA) vaccine antibody titers in case of treatment with anti-CD20 monoclonal antibodies within the last 12 months [82]. A recent study in patients with autoimmune disorders and a history of anti-CD20 directed therapies observed a cellular vaccine response after mRNA vaccination in 20% of patients [111]. However, the proportion of patients with cellular vaccine response was significantly lower than observed among healthy controls (75%) [111]. Of note, most patients had immunosuppressive co-medication, in particular steroids in nearly half of patients [111]. In contrast, in a small study of multiple sclerosis patients on active monotherapy with anti-CD20 monoclonal antibodies, all patients developed a CD4+ and CD8+ T cell response to vaccination with COVID-19 mRNA-based vaccines, which suggests that, in CLL, the underlying hematological disease might also play a role [60].

In a further cohort study on lymphoma patients with a history of anti-CD20 treatment, a cellular response to COVID-19 vaccination, mostly mRNA-based, was observed in 58% of evaluable patients with no association between T cell response and time to last anti-CD20 therapy [112]. A larger study evaluating response to COVID-19 mRNA vaccines in a cohort of cancer patients reported a SARS-CoV-2-specific T cell reactivity in 45% of patients with hematological cancer and 34% of patients with anti-CD20 therapies and notably observed no significant association between anti-CD20 therapy versus other types of therapies and T cell reactivity [31].

Following cellular therapy with anti-CD19 CAR T cells, a positive cellular response to BNT162b2 vaccination was observed in 50% of patients in a small prospective cohort trial, including patients without humoral response and complete B cell aplasia [62]. In multiple myeloma patients receiving either one of the COVID-19 mRNA vaccines or the vector-based vaccine AZD1222, treatment with anti-CD38 monoclonal antibodies or anti-BCMA ADCs was associated with significantly reduced humoral vaccine response [113,114]. With respect to cellular vaccine response, a small study on myeloma patients following COVID-19 mRNA vaccination found a similar SARS-CoV-2 specific CD4+ and CD8+ T cell response in seropositive myeloma patients and healthy controls, while seronegative myeloma patients showed significantly reduced CD4+ T cell responses [115]. In particular, active treatment with anti-CD38 monoclonal antibodies or anti-BCMA bispecific antibodies was associated with decreased CD4+ T cell responses, while patients treated with anti-BCMA CAR T cells mounted similar CD4+ T cell responses compared to myeloma patients receiving other therapies [115].

### 4.5. Anti-Cancer Treatments: Chemotherapy, Immune Checkpoint Blockade and Small Molecules

As vaccination studies tend to report mainly on humoral vaccine response, data on cellular vaccine response in specific subgroups of the large variety of anti-cancer therapies are scarce and often hampered by small sample size and insufficient statistical power. Given the unique challenge of the COVID-19 pandemic and the observation of strong cellular immune response in COVID-19 vaccines [56,64], recently, several studies on COVID-19 vaccination in cancer patient also report on cellular vaccine response and help to elucidate this issue.

The prospective VOICE trial reported on response to two-dose COVID-19 mRNA vaccination with mRNA-1273 in solid tumor patients [25]. A SARS-CoV-2 spike-specific IFNγ T cell response at four weeks after dose 2 was observed in 67% of evaluable patients with chemotherapy, 66% with immunotherapy, and 53% with chemoimmunotherapy, compared to 69% in controls [25]. Of particular interest, a cellular vaccine response was observed in 43% of serological non-responders and 47% of suboptimal responders among cancer patients [25].

To assess the impact of booster vaccination of mRNA-based COVID-19 vaccines in cancer patient on active immunosuppressive therapy, by the vast majority, cytotoxic chemotherapy, a prospective cohort study evaluated humoral and cellular response following one to three doses [34]. While the control cohort already showed a significant increase in INFγ producing T cells after the first dose compared to pre-vaccination, in cancer patients, a clear four-fold increase was only observed after the second dose, with T cell frequencies still significantly lower than observed in healthy controls [34]. Notably, while humoral vaccine response significantly improved after a third dose in cancer patients, no overall increase in T cell response could be observed following booster immunization [34]. This contrasts with a recent report from the UK showing that after a third vaccination dose specific T cell response increased from around 35% to around 73% in patients with solid tumors and hematological malignancies [108]. In particular, the T cell responses were more robust against variants of concern, therefore possibly providing a more durable protection [8,108].

A cohort study on the immunogenicity of COVID-19 vaccine BNT162b2 in cancer patients receiving immune checkpoint inhibitors either targeting programmed cell death protein 1 (PD-1) or programmed death-ligand 1 (PD-L1) showed a spike-specific T cell response, assessed by ELISpot, in 72% of patients after one dose and in 92% after the second dose [116]. Both CD4+ and CD8+ T cells were elicited by vaccination, and no significant correlation between increase in spike-specific T cells and time interval between start of immunotherapy and vaccination was observed [116]. A small study on patients with chronic myeloid leukemia under active treatment with tyrosine kinase inhibitors also reported highly promising humoral and cellular response to one dose of COVID-19 vaccine BNT162b2 with polyfunctional T cell responses observed in 80% of evaluable patients [117].

In summary, recent COVID-19 vaccination studies in patients with cancer have shown a depth of analysis that has not been reported before and provides important and novel evidence supporting the essential role of the T cellular response for protection against vaccine-preventable disease.

## 5. Development of Novel Vaccines

In light of the essential role of T cell response to vaccination, vaccines that specifically target this axis are needed for immunocompromised patients. Currently, such developments are under investigation. A novel COVID-19 vaccine consisting of several viral peptides and a TLR-1/2 agonist as adjuvant has shown promising activity in eliciting T cell responses [118,119]. Importantly, in first clinical trials, the vaccine CoVac1 is well tolerated and induces T cells to all viral strains tested so far [53]. This implies that protection from COVID-19 will be robust and durable even after antigenic shift caused by VOC. Further, it has been shown that heterologous prime-boost schedules favor T cell responses and may therefore be reasonable approaches for immunocompromised patients [120].

## 6. Conclusions and Outlook

Cellular response, in particular T cell response, is often more reliably induced in cancer patients than the antibody response. It may be a suitable correlate of protection and, further, more robust against the antigen drift and possible ensuing immune escape resulting from pathogen mutations. Novel vaccines developed especially for patients with impaired immune response may take this into account.
